# Wild Duck (*Anas platyrhynchos*) as a Source of Antibiotic-Resistant *Salmonella enterica* subsp. *diarizonae* O58—The First Report in Poland

**DOI:** 10.3390/antibiotics11040530

**Published:** 2022-04-15

**Authors:** Joanna Pławińska-Czarnak, Karolina Wódz, Lidia Piechowicz, Ewa Tokarska-Pietrzak, Zbigniew Bełkot, Janusz Bogdan, Jan Wiśniewski, Piotr Kwieciński, Adam Kwieciński, Krzysztof Anusz

**Affiliations:** 1 Department of Food Hygiene and Public Health Protection, Institute of Veterinary Medicine, Warsaw University of Life Sciences, Nowoursynowska 159, 02-776 Warsaw, Poland; janusz_bogdan@sggw.edu.pl (J.B.); jan_wisniewski1@sggw.edu.pl (J.W.); krzysztof_anusz@sggw.edu.pl (K.A.); 2 Laboratory of Molecular Biology, Vet-Lab Brudzew, Turkowska 58c, 62-720 Brudzew, Poland; karolina.wodz@labbrudzew.pl (K.W.); vetlab@interia.pl (P.K.); kwiecinski@vetlabbrudzew.pl (A.K.); 3 Department of Medical Microbiology, Faculty of Medicine, Medical University of Gdańsk, Dębowa 25, 80-204 Gdańsk, Poland; lidiap@gumed.edu.pl (L.P.); etok@gumed.edu.pl (E.T.-P.); 4 Department of Food Hygiene of Animal Origin, Faculty of Veterinary Medicine, University of Life Sciences in Lublin, Akademicka 12, 20-950 Lublin, Poland; zbigniew.belkot@up.lublin.pl

**Keywords:** *Salmonella enterica* subsp*. diarizonae*, antibiotic-resistant *Salmonella*, wild duck

## Abstract

The “One Health” approach increasingly demonstrates the global spread of pathogenic microorganisms and their antimicrobial resistance in the environment, both in animals and humans. *Salmonella enterica* subsp. *diarizonae* is nowadays very often isolated from cold-blooded reptiles to a lesser extent from sheep, but unfortunately more and more often from humans. However, there are a few studies describing the isolation of *Salmonella enterica* subsp. *diarizonae* from migratory wild birds. The mallard duck (*Anas platyrhynchos*), a wild animal that traverses the continent of Eurasia, can be an excellent indicator of the spread of intestinal microbes as well as their resistance to antibiotics. This is the first report of the *Salmonella enterica* subsp. *diarizonae* detection in Poland in a migrating mallard duck. This research presented the identification difficulties associated with the isolation of *Salmonella enterica* subsp. *diarizonae* using three different biochemical tests and advanced serology tests. At the same time, we detected very high antimicrobial resistance in the isolated strain. By using the minimum inhibitory concentration (MIC) method, it was found that the isolated strain of *S. enterica* subsp. *diarizonae* has high antibiotic resistance against 14 of the 33 tested antimicrobials agents. The resistance genes that have been identified in *S. enterica* subsp. *diarizonae* include *aadA*, *strA/strB*, and *bla*_TEM_.

## 1. Introduction

Salmonellosis is an important cause of water-borne [1] and food-borne epidemics in humans. In the European Union, salmonellosis is the second most frequently reported human gastrointestinal infection right after campylobacteriosis. In 2021 a total of 90,105 human salmonellosis cases were reported by EU EFSA [2]. People contract *Salmonella* not only from animal origin foods but also from eating contaminated vegetables and fruits or from their pets. Now there are more and more reports of human diseases caused by *Salmonella enterica* subsp. *diarizonae* [3]. *Salmonella enterica* subsp. *diarizonae* is a subspecies IIIb of six within *Salmonella enterica* spp. It is most often described as a Gram-negative rod infecting cold-blooded animals, including reptiles kept as pets [4,5]. However, *Salmonella enterica* subsp. *diarizonae* was also isolated in Norway and Switzerland from sheep [6,7] and Greece [8]. Chatzopoulos et al. showed that *Salmonella enterica* subsp. *diarizonae* is an opportunistic gastrointestinal pathogen in lamb [9].

Thus, the spread of *Salmonella enterica* subsp. *diarizonae* in the environment is only a matter of time.

Taking into account the well-known concept of caring for “One Health” approach, which relates to both the protection of human and animal health and considering the impact of the environment on humans and animals, the WHO poses the problem of antibiotic-resistant pathogens as one of the main challenges the health care of the 21st century is facing. The level of antibiotic resistance of bacteria in the environment can be indirectly tested by pathogens isolated from wild animals, such as migratory birds. The mallard duck (*Anas platyrhynchos*), also known as the wild duck, is commonly found in Poland and Europe. It is a migrating bird; therefore, it is also found in North America and Asia. It lives in areas of wild nature and urban agglomerations in the vicinity of water reservoirs [10]. Mallards can lead a sedentary or wandering/migratory lifestyle; therefore, it is an excellent bioindicator of the occurrence of pathogens and drug-resistant bacteria in the local and cross-border environment.

The aim of this study was to serologically and biochemically identify and determine the antibiotic resistance of *Salmonella* spp. isolated from mallard ducks.

## 2. Results

In the examined 17 mallard ducks (nine male and eight female), no pathological changes were observed in the muscle, liver, and intestine. From all the examined internal organs, only seven strains of *Salmonella* spp. from the intestines have been isolated. All strains belonged to *Salmonella enterica* spp. Six were classified as *Salmonella enterica* subsp. *enterica* (two strains of *S.* Derby and four strains of *S.* Enteritidis) and one strain presumptive *Salmonella enterica* subsp. *diarizonae*. A strain suspected of being *Salmonella enterica* subsp. *diarizonae* was checked for further analysis because, so far, it has not been isolated from mallard ducks in Poland. The isolated strain of *Salmonella* spp. in the applied biochemical tests showed diversified results. Common positive reactions from the three tests include H_2_S production, D-glucose, D-mannitol, and lysine decarboxylase for VITEK and Lab-made beta-galactosidase, fermentation/glucose, D-maltose, and D-trehalose, and for API 20 E and Lab-made tests—arabinose (other results were presented in Supplementary Materials Table S1).

Therefore, we obtained the classification of various *Salmonella enterica* spp. In the API 20E test, the strain was designated as 89.6% *Salmonella* spp., 10.3% *Salmonella enterica* subsp. *arizonae* and VITEK in 99% of *Salmonella enterica* subsp. *diarizona* (detailed reactions in individual tests are presented in Supplementary Materials Table S1).

### 2.1. Serological Identification

As a result of the serotyping, the tested isolate was identified as *S. enterica* subsp. *diarizonae* O:58 group strain. This result requires confirmation by the WHO Collaborating Centre for Reference and Research on Salmonella, Institut Pasteur (Paris, France).

### 2.2. Antimicrobial Susceptibility Testing

The following chemotherapeutic agents (MERLIN Diagnostika GmbH, Bremen, Niemcy) were included in our study: Ampicillin (AM), Amoxicillin/Clavulanic acid (AMC), Cefalexin (CN), Cefalotin (CF), Ceftriaxone (CFP), Cefequinome (CEQ), Ceftiofur (CFT), Enrofloxacin (ENR), Florfenicol (FFC), Flumequine (UB), Gentamicin (GM), Imipenem (IPM), Marbofloxacin (MRB), Neomycin (NEO), Polymixin B (PB), Tetracycline (TE), and Trimethoprim/Sulfamethoxazole (SXT) and additional antibiotics, Cefalotin (CF), cefoperazone (CFP), Imipenem (IPM), Flumequine (UB), Marbofloxacin (MRB), Tetracycline (TE), Polymixin B (PB), and Trimethoprim/Sulfamethoxazole (SXT).

The *S. enterica* subsp. *diarizonae* O: 58 group strain was resistant to 14 antimicrobials (CFX-CPH-CLO-PG-NAF-GEN-NEO-STR-UB-ERY-TYL-LIN-TIA-TYLV) belonging to five classes of antimicrobials agents (β-lactams, aminoglycosides, fluoroquinolones, macrolides, and lincosamides) and florfenicol, tiamulin, tylvalosin. Resistance to individual antibiotics is shown in Table 1.

### 2.3. Detection of Antimicrobial Resistance Genes (ARGs) by Multiplex PCR

**Figure 1:**
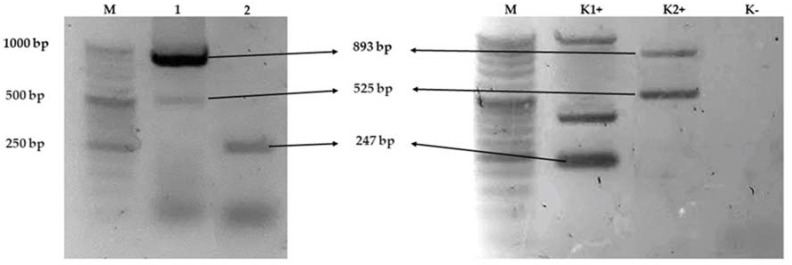
Agarose gel (2%) electrophoresis showing the amplification of multiplex PCR line 1 *strA/strB* (893 bp) and *aadA* (525 bp), multiplex PCR line 2 *bla_TEM_* (247 bp) of *Salmonella enterica* subsp. *diarizonae* O58. Notes: line M DNA marker, line K1+ *bla_TEM_* (247 bp), and line K2+ positive control genes *sul2* (721 bp), *aadA* (525 bp), as positive controls were *Klebsiella pneumoniae* and *E. coli* strains isolated from poultry and cattle. Lane K—no template (negative control). Abbreviations: M—DNA marker (DNA Marker 2+, (50–1000 bp, A&A Biotechnology Gdańsk, Poland); bp—base pairs.

Antimicrobial resistance genes present in *Salmonella enterica* subsp. *diarizonae* O:58 isolated from wild ducks (*Anas platyrhynchos*) (Figure 1). The isolate harboured the *aadA*, *strA/strB*, *bla_TEM_* genes, while *aphA1*, *aphA2*, *aadB*, *tetA*, *tetB*, *sul1*, *sul2*, *bla_SHV_*, *bla_CMY-2_* were absent.

## 3. Discussion

Mallard duck belongs to the order of the *Anseriformes* during foraging. It filters the surface of the water reservoir, which makes them vulnerable to collecting numerous contaminants with food. They are also often found in large flocks of the same species and groups with other waterfowl, which may additionally predispose them to cross infections. The area where the duck was collected for research is a typical field circuit with a large number of water reservoirs, rivers, and streams. It is a very favourable habitat for waterfowl that frequently use the area, including sedentary as well as migratory populations. The northern duck, as the migratory population, is characterized by a higher body weight compared with the permanent population. Numerous studies on mallard migration indicate that ducks come to Poland from Finland, Russia, Belarus, and Ukraine, and often from Western Europe, countries such as France, Germany, Austria, and the Czech Republic [11,12,13]. It can be said with a high probability that the ducks described in the research came from the eastern part of Europe, including the countries of Belarus, Ukraine, and Russia. Thus, migration may play a role in the transmission of *Salmonella* spp. and antibiotic-resistant bacteria. Previously, greylag goose was described as the possible vector of *Salmonella enterica* subsp. *diarizonae* 14:k:z53 in Norway [14].

Table S1 shows the comparison between VITEK2 and API20E results, respectively. This table shows the different points and similarities of the results in the same samples when applying the other methods. This demonstrates that the commonly used API system does not allow for unambiguous identification of *Salmonella* spp., other than *Salmonella enterica* subsp. *enterica*.

The drawback of the traditional phenotypic method is that it requires the availability of more than 150 specific antisera and well-trained, experienced personnel to correctly interpret the results. Consequently, it is not possible for all laboratories to carry out this method in-house, and often, laboratories have to send the isolates to a national reference laboratory or an expert laboratory. This process can significantly delay the time of obtaining results. In terms of performance, the method may give false positive reactions due to weak or nonspecific agglutination. Autoagglutination or loss of antigen expression, as observed for rough and nonmotile isolates, results in unidentified serotypes [15,16]. Our research clearly shows difficulties in classical biochemical and serological identification of *Salmonella* ssp., especially between IIIa and IIIb. Data on the isolation of *Salmonella enterica* subsp. *diarizonae* strains in Poland are limited. According to the National Salmonella Center from 1995–2007 *Salmonella enterica* subsp. *diarizonae* was identified sporadically, much less than 1% [15]. Serological analysis of these *Salmonella enterica* subsp. *diarizonae* strains occurring in Poland presented several different serovars. Six serovars that belonged to groups O35, O38, O48, O58, O60, and O61 have been identified, mainly in animals and humans. Amongst them, the serovar *Salmonella enterica* subsp. *diarizonae* 58:z52:z35, i.e., S. IIIb (58:z52:z35), was detected twice in 1998 (in the internal organs of the biting adder *Bitis arietans* and in the faeces of the *Canadian beaver Castor canadensis*). They were lactose-positive strains. In the same year, single isolation of the strain S. IIIb (48:k:z57) was obtained from the water (wild bathing area, river Raba, near Myślenice). The strain was lactose negative, ONPG positive after 2 h and presented a hitherto unknown antigenic formula. Strains of *Salmonella enterica*. subsp. *diarizonae* serovars *S*. IIIb (48: k:z53) and *S*. IIIb (61: k:1,5,7) were also isolated from two patients requiring hospitalization (child’s faeces, adult cerebrospinal fluid) [15].

Amongst *Salmonella* strains originating mainly from the environment, food and isolates from animals which were sent to the National Salmonella Center in Poland in the years 2008–2020, fourteen strains of *Salmonella enterica* subsp. *diarizonae* were identified (<1%). They represented different serovars of groups O38, O47, O48, O50, and O61. Strains of serovar *S*. IIIb (38:r:z) were isolated from food and the environment. Seven serovars *S*. IIIb (47:k:z35), *S*. IIIb (48:k:z53), *S*. IIIb (50:z:z52), *S*. IIIb (50:z52:z35), *S*. IIIb (50:r:z35), *S*. IIIb (61:c:z35), and *S*. IIIb (61:k:1,5) were found in human (unpublished study, Tokarska-Pietrzak E. and Piechowicz L.). Four serovars: *S*. IIIb (48:i:z), S. IIIb (61:r:z), *S*. IIIb (61:c:z35), and *S*. IIIb (65:k:z), were identified in animals. In 2021, one *Salmonella enterica* subsp. *diarizonae* strains were isolated from a duck. It belonged to group O58 and represented a serovar that was isolated in wild mallard ducks in Poland for the first time.

Because of *Salmonella* spp. significance to the poultry industry and the risk of alimentary infections in humans, a large number of studies have been carried out on its epidemiology in wild birds such as mallard ducks. Thus, there are guidelines within the poultry industry to avoid or minimize contact between wild birds and domestic poultry, especially with ducks and geese bred in an open system. Wild birds are not considered a direct source of infection for livestock but rather a source of feed contamination amongst farm animals. The resistance of bacteria to one or more antibiotics poses a risk for human and animal health. Ducks and other wild birds may be colonized by antibiotic-resistant bacteria present in their environment. It can be hypothesized that wild birds can act as vectors or amplifiers, which carry resistant bacteria to livestock.

The results of the phenotypic resistance test indicated that *Salmonella enterica* subsp. *diarizonae* O:58 isolated from mallard ducks could be categorized as MDR, that is, bacteria exhibiting resistance to one or more antibiotics from three or more classes of antibiotics [17,18].

The isolate harboured the *aadA*, *strA/strB*, major genes for resistance to streptomycin, but not for gentamycin (*aadB*) and neomycin (*aphA1*, *aphA2*), which belong to aminoglycosides. In addition, we found the *bla*_TEM_ gene that belongs to ESBL-encoding genes associated with extended-spectrum resistance to β-lactams in multidrug-resistant strains of Gram-negative bacteria such as *Klebsiella pneumoniae*. However, the strains were phenotypically sensitive to ampicillin, probably because of the gene being silenced. ESBL-encoding genes are usually localized on large plasmids that simplify their spread amongst gram-negative rods via conjugation. What is more, these plasmids may carry other genes associated with resistance to other classes of antibiotics. Because of the plasticity of *Salmonella*, its adaptability, and the development of mechanisms of resistance to antibiotics using genetic strategies such as gene mutations or horizontal transfer of resistance genes [19], the presence of the *bla*_TEM_ gene seems to be alarming.

The *aadB* gene coding resistance to gentamycin, and *aphA1*, *aphA2* to neomycin, were not found in the present study. However, the strain was resistant to this antibiotic. This resistance to gentamycin may be mediated by other resistance genes such as *grm* [20], which was not evaluated in this study.

The next step of the research will be the serological analysis of the isolated strain *Salmonella enterica* subsp. *diarizonae* O:58 at the Pasteur Institute in Paris and NGS Whole Genome Sequencing

## 4. Materials and Methods

The material for the research was fragments of liver, muscles, and intestines obtained from 17 shot mallard ducks (*Anas platyrhynchos*) in nature from hunting grounds in Lubelszczyzna in Poland. The ducks were harvested in accordance with the Polish hunting law [10] by shooting during the hunting season, which lasts from 1 September to the end of December 2021. The mallard (*Anas platyrhynchos*) is on the list of game species in accordance with the Regulation of the Minister of the Environment on establishing the list of game species [21]. After harvesting, all uneviscerated birds were packed in a foamed polystyrene refrigerated box and transported to the laboratory at 4 °C.

### 4.1. Salmonella spp. Isolation and Identification

*Salmonella* spp. from all samples were isolated in accordance with PN-EN ISO 6579-1:2017-04 Microbiology of the food chain—Horizontal method for the detection, enumeration, and serotyping of Salmonella—Part 1: Detection of Salmonella spp. [22].

Samples were suspended in a ninefold volume of buffered peptone water (BPW GRASO, Starogard, Poland) for each 1 g of sample (5 g of liver, 5 g of intestines and 25 g of muscle) in sterile stomacher bags (Whirl-Pak, NASco, Madison, WI, USA). Then they were placed in a stomacher and crushed for 2 min. The selective proliferation of *Salmonella* spp. was carried out using modified semisolid Rappaport-Vassiliadis (MSRV) agar (GRASO) and Muller–Kauffmann tetrathionate-novobiocin (MKTTn) broth (GRASO). Two selective enrichment media, xylose lysine deoxycholate agar (XLD; GRASO) and Salmonella chromagar agar (CHROMagar Salmonella PLUS; GRASO), were used as described in Pławińska-Czarnak et al. (2021) [23]. Salmonella suspect colonies were transferred to nonselective nutrient agar (GRASO) to obtain the pure culture for further biochemical and to a semisolid medium by Garda (GRASO) for further serological tests.

#### 4.1.1. *Salmonella* spp. Identification with Molecular Biology Methods

A real-time PCR method based on the detection of genes specific for *Salmonella* spp. was used to confirm presumptive identification. DNA for real-time PCR was extracted from bacterial cells using a commercial Kylt^®^ DNA Extraction-Mix II (AniCon, Hoeltinghausen, Germany). Real-time PCR to detect *Salmonella* was performed according to the manufacturer’s instructions (AniCon, Hoeltinghausen, Germany) using Applied Biosystems 7500 Fast Real-Time PCR System (Thermo, Waltham, MA, USA).

#### 4.1.2. Biochemical Strain Identification

Biochemical tests were also conducted with the use of homemade media inoculated with bacteria grown on nutrient agar, incubated for 24 h (with the exception of dulcitol, inositol, and rhamnose fermentation tests, gelatine hydrolysis test, and d-tartrate test—in case the results were negative after 24 h, the incubation was prolonged up to 5 days). The tests with negative results were verified and repeated.

For biochemical identification of the strains, the VITEK^®^ 2 GN cards and Api20E (BioMérieux, Craponne, France) were used according to the manufacturer’s instructions. Biochemical tests were also done with the use of lab-made media, inoculated with bacteria grown on nutrient agar, incubated for 24 h (with the exception of dulcitol, inositol, and rhamnose fermentation tests, gelatine hydrolysis test, and d-tartrate test—in case while the results were negative after 24 h, the incubation was prolonged up to 5 days) according to Załęska et al. (1969) [24]. The tests with negative results were verified and repeated.

#### 4.1.3. Serological Typing

Serological typing of *Salmonella* for the detection of somatic and flagellar antigens was performed using the slide agglutination test. The polyvalent and monovalent *Salmonella* anti-O and anti-H diagnostic sera were used (Statens Serum Institut, Copenhagen, Denmark; BIOMED, Kraków, Poland; Immunolab, Gdynia, Poland). The results of the antigen determination were used for the final serological characterization using the White–Kauffmann–Le Minor scheme [25,26]. A 24 h culture of the tested strain on nutrient agar medium was used for the tests (BioMaxima, Lublin, Poland).

### 4.2. Antimicrobial Susceptibility Testing

The 8 classes of antimicrobials agents (β-lactams, aminoglycosides, polymyxins, fluoroquinolones, tetracyclines macrolides, lincosamides, and sulfonamide) as well as florfenicol, tiamulin, and tylvalosin were used for the antimicrobial susceptibility test.

Antimicrobial susceptibility was assessed by determining the MIC values using a 96 well MICRONAUT Special Plates with antimicrobials: AMX/CL—amoxicillin and clavulanic acid; AMX—amoksycylina; CFQ—cefquinome; CFTI—ceftiofur; CFX—cephalexin, CLO—cloxacillin; COL—colistin; CPH—cefapiryna; DOX—docycycline; ENR—enrofloxacin; ERY—erythromycin; FLR—florfenicol; GEN—gentamicin; LIN—lincomycin, LIN/SP—lincomycin/specinicin; NAF—nafcillin; NEO—neomycin; NOR—norfloxacin; OXY—oxytetracycline; PG—benzylpenicillin; STR—streptomycin; TR/SMX—trimethoprim-sulfamethoxazole; TIA—tiamulin; TYL—tylosin; TYLV—tylvalosin (MERLIN Diagnostika GmbH, Bremen, Niemcy). To analyze MIC patterns of *S. enterica* subsp. *diarizonae* Merlin MICRONAUT (MERLIN Diagnostika GmbH, Bremen, Niemcy) was used. The MICs were interpreted according to the Clinical and Laboratory Standards Institute (CLSI) and FDA breakpoints [27].

Minimum Inhibitory Concentrations (MICs) of Amoxicillin/Clavulanic acid (AMC), Ampicillin (AM), Cefalexin (CN), Cefalotin (CF), Ceftriaxone (CFP), Cefequinome (CEQ), Ceftiofur (CFT), Enrofloxacin (ENR), Florfenicol (FFC), Flumequine (UB), Gentamicin (GM), Imipenem (IPM), Marbofloxacin (MRB), Neomycin (N), Polymixin B (PB), Tetracycline (TE), and Trimethoprim/Sulfamethoxazole (SXT) and additional antibiotics, Ampicillin (AM), Cefalotin (CF), cefoperazone (CFP), Imipenem (IPM), Flumequine (UB), Marbofloxacin (MRB), Tetracycline (TE), Polymixin B (PB), Trimethoprim/Sulfamethoxazole (SXT) were assessed by AST-GN 96 CARD and VITEK2 system. The AST card is essentially a miniaturized and abbreviated version of the doubling dilution technique for MICs determined by the microdilution method [28].

### 4.3. Detection of Antimicrobial Resistance Genes (ARGs) by Multiplex PCR

Mueller–Hinton agar was used to culture the bacterial isolates overnight at 35 °C. Bacterial DNA isolation was performed using a standard bacterial DNA isolation Kylt^®^ DNA Extraction-Mix II (Anicon, Emstek, Germany).

Twelve resistance genes (*aadA*, *aadB*, *strA/strB*, *aphA1*, *aphA2*, *tetA*, *tetB*, *sul1*, *sul2*, *bla* *_TEM_*, *bla_SHV_*, and *bla_CMY-2_*) were analyzed by conventional PCR using specific primer pairs). The primer sequences predicted PCR product sizes and references shown in Table 2.

Multiplex PCR protocols were established to screen for genes linked with antibiotic resistance of *S. enterica* subsp. *diarizonae*. Each PCR reaction contained 3 μL of DNA (20 ng), 12.5 μL of DreamTaq Green 2 × Master Mix (Thermo Fisher Scientific, Waltham, MA, USA), 20 pmol of each of the forward and reverse primers (Genomed, Warsaw, Poland), and nuclease-free water to a final volume of 25 µL. PCR conditions in all Multiplex were as follows: 1 cycle at 95 °C for 3 min for an initial denaturation, followed by 35 cycles of denaturation for 30 s at 95 °C, primer annealing for 30 s at temperature described in Table 2, primer extension for 1 min at 72 °C, and a final extension for 7 min at 72 °C. All reactions were performed on GeneAmp PCR System 2700 Thermocycler (Applied Biosystems, Waltham, MA, USA). PCR primers, product size and sources are described in Table 2. The PCR products were visualized in 2% agarose gels containing Ethidium Bromide (Invitrogen, Waltham, MA, USA) under UV radiation. PCR followed thermal cycling according to Matsui et al. (2021) [30].

## 5. Conclusions

Our research showed that *Salmonella enterica* subsp. *diarizonae* does not cause pathological changes in the internal organs of mallard ducks (*Anas platyrhynchos*), so there is a very high probability of transmitting the pathogen over long distances in the intestinal trackt. On the other hand, studies have shown very serious difficulties in the routine diagnosis of *Salmonella enterica* subsp. *diarizonae*. The use of standard biochemical tests (such as API20E) and commercially available sera carries a high risk of confusing the strain with closely related *Salmonella enterica* subsp. *arizonae*. The fact of the occurrence of multidrug resistance in the studied strain is very disturbing.

The results of this study suggest that wild waterfowl may serve as a significant vector of antibiotic-resistant bacteria and may have a crucial impact on public health. To our knowledge, this is the first case of detection of *S. enterica* subsp. *diarizonae* in wild duck and its biochemical and antibiotic resistance pattern analysis. A better understanding of the factors that potentially contribute to the spreading of resistant zoonotic pathogens can improve the tools used by public health specialists to control antimicrobial resistance. More studies with larger numbers of samples, and DNA sequencing of bacterial strains, are needed to prove the hypothesis about the exact routes of transmission of antibiotic-resistance genes and *S. enterica* subsp. *diarizonae* itself. The prevalence of migrated mallard ducks around the world indicates that they may be a vector that transfers antimicrobial resistance genes from countries with high antibiotic use to countries with programs to reduce the use of chemotherapeutic agents. This can have serious implications for the health of wildlife, livestock, and humans.

## Figures and Tables

**Table 1:** Antimicrobial resistance of the *Salmonella enterica* subsp. *diarizonae* O:58 isolate from mallard ducks.

Antibiotic	Minimal Inhibitory Concentration, μg/mL	Sensitivity
ampicillin	≤2	S
amoxicillin	2	S
amoxicillin and clavulanic acid	≤2	S
cefalexin	≥8	R
cefalotin	≤2	S
cefapirin	≥8	R
cefoperazone	≤4	S
ceftiofur	≤2	S
cefquinome	≤2	S
cloxacillin	>2	R
penicillin G	8	R
nafcillin	>2	R
imipenem	≤0.25	S
gentamicin	≥16	R
neomycin	≥8	R
streptomycin	≥32	R
colistin	≤2	S
polymixin B	≤2	S
enrofloxacin	≤0.5	S
flumequine	≥32	R
marbofloxacin	≤0.25	S
norfloxacin	≤1	S
doxycycline	≤2	S
oxytetracycline	≤2	S
tetracycline	≤1	S
erythromycin	>0.5	R
tylosin	> 1	R
florfenicol	4	I
lincomycin	≥8	R
lincomycin/spectinomycin	≤8	S
trimethoprim–sulfamethoxazole	≤2	S
tiamulin	>16	R
tylvalosin	>4	R

Labelling as resistant (R), intermediate (I) or susceptible (S) for a specific antimicrobial is indicated in the rows.

**Table 2:** Primers sequences for detection of antimicrobial resistance genes in the *Salmonella* spp. isolate and multiplex PCR annealing temperature [29,30].

Multiplex PCR	Gene	Primer Sequences 5′–3′	Annealing Temperature	Product Size (bp)
1	*aadA*	F: GTG GAT GGC GGC CTG AAG CC R: AAT GCC CAG TCG GCA GCG	63 °C	525 bp
1	*strA/strB*	F: ATGGTG GAC CCT AAA ACT CT R: CGT CTA GGA TCG AGA CAA AG	63 °C	893 bp
2	*aphA1*	F: ATG GGC TCG CGA TAA TGT C R: CTC ACC GAG GCA GTT CCA T	55 °C	634 bp
2	*aphA2*	F: GAT TGA ACA AGA TGG ATT GC R: CCA TGA TGG ATA CTT TCT CG	55 °C	347 bp
2	*aadB*	F: GAG GAG TTG GAC TAT GGA TT R: CTT CAT CGG CAT AGT AAA AG	55 °C	208 bp
3	*tetA*	F: GGC GGT CTT CTT CAT CAT GC R: CGG CAG GCA GAG CAA GTA GA	63 °C	502 bp
3	*tetB*	F: CGC CCA GTG CTG TTG TTG TC R: CGC GTT GAG AAG CTG AGG TG	63 °C	173 bp
4	*sul1*	F: CGG CGT GGG CTA CCT GAA CG R: GCC GAT CGC GTG AAG TTC CG	66 °C	433 bp
4	*sul2*	F: CGG CAT CGT CAA CAT AAC CT R: TGT GCG GAT GAA GTC AGC TC	66 °C	721 bp
5	*bla*_TEM_	F: TTAACTGGCGAACTACTTAC R: GTCTATTTCGTTCATCCATA	55 °C	247 bp
5	*bla*_SHV_	F: AGGATTGACTGCCTTTTTG R: ATTTGCTGATTTCGCTCG	55 °C	393 bp
5	*bla*_CMY-2_	F: GACAGCCTCTTTCTCCACA R: TGGACACGAAGGCTACGTA	55 °C	1000 bp

Abbreviations: bp—base pairs.

## Data Availability

The data presented in this study are available in Supplementary Materials.

## Supplementary Materials

The following supporting information can be downloaded at: https://www.mdpi.com/article/10.3390/antibiotics11040530/s1, Table S1: a summary of the results of biochemical reactions for the isolated strain of *Salmonella* spp. in the VITEK test, API-20E, and individual biochemical lab-made tests.
