# Supplementary material for: Wild Duck (Anas platyrhynchos) as a Source of Antibiotic-Resistant Salmonella enterica subsp. diarizonae O58—The First Report in Poland
Source: Antibiotics (Basel). 2022 Apr 15;11(4):530. doi: 10.3390/antibiotics11040530 (PMC9024997; doi:10.3390/antibiotics11040530)
Supplement: Supplementary file 1 [file antibiotics-11-00530-s001.zip › antibiotics-1675549-supplementary.pdf]

Supplemental materials Table S1. A summary of the results of biochemical reactions for the isolated strain of *Salmonella enterica* subsp. *diarizonae,* O58 from mallard duck (*Anas platyrhynchos*) from Poland, in the VITEK test, API-20E and individual biochemical lab-made tests

**Reactions/ Enzymes**	**VITEK2** *Salmonella enterica* **spp.** ***diarizonae*** (99% pobability)	**API 20E** **89,6%** ***Salmonella*** **spp/ 10,3%** ***Salmonella enterica*** **ssp.** ***arizonae***.	**Individual biochemical lab-made tests**
BGAL	+	-	+
**H2S**	**+**	**+**	**+**
**dGLU**	**+**	**+**	**+**
**dMAN**	**+**	**+**	**+**
URE	-	-	–
dSOR	+	-	not included
SAC	-	-	–
CIT	-	-	+
ODC	+	+	not included
**LDC**	**+**	**+**	**+**
APPA	-	not included	not included
ADO	-	not included	not included
PyrA	-	not included	not included
IARL	-	not included	not included
dCEL	-	not included	not included
BNAG	-	not included	not included
AGLTp	-	not included	not included
GGT	-	not included	not included
OFF	+	not included	+
BGLU	-	not included	not included
dMAL	+	not included	+
dMNE	+	not included	not included
BXYL	-	not included	not included
BAlap	-	not included	not included
PrpA	-	not included	not included
LIP	-	not included	not included
PLE	-	not included	not included
TyrA	-	not included	not included
dTAG	-	not included	not included
dTRE	+	not included	+
MNT	-	not included	+
5KG	-	not included	not included
lLATk	-	not included	not included
AGLU	-	not included	not included
SUCT	-	not included	not included
NAGA	-	not included	not included
AGAL	+	not included	not included
PHOS	-	not included	not included

GlyA	-	not included	not included
lHISa	-	not included	not included
CMT	+	not included	not included
BGUR	+	not included	not included
O129R	-	not included	not included
GGAA	-	not included	not included
lMLTa	-	not included	not included
ELLM	-	not included	not included
lLATa	-	not included	not included
ADH	not included	+	not included
TDA	not included	-	not included
IND	not included	-	–
VP	not included	-	not included
GEL	not included	-	–
INO	not included	-	–
RHA	not included	+	–
MEL	not included	+	not included
AMY	not included	-	not included
ARA	not included	+	+
LAC	not included	not included	–
SAL	not included	not included	–
XYL	not included	not included	+
DUL	not included	not included	–
dTAR	not included	not included	–
MUC	not included	not included	–

Common reaction for VITEK, API 20 E and Lab-made tests: BGAL/ONPG - beta-galactosidase, H2S - H2S production, dGLU/GLU - D-glucose, dMAN/MAN - D-mannitol, URE – urease, dSOR/SOR - D-sorbitol (not includet Lab-made), SAC - saccharose/sucrose, CIT - citrate (sodium), ODC - ornithine decarboxylase (not includet Lab-made), LDC - lysine decarboxylase,

VITEK: APPA - Ala-Phe-Pro-arylamidase, ADO – adonitol, PyrA - L-pyrrolydonyl-arylamidase, IARL - L-arabitol, dCEL - D-cellobiose, BNAG - beta-n-acetyl-glucosaminidase, AGLTp - Glutamyl Arylamidase pNA, GGT - gamma-glutamyl-transferase, OFF - fermentation/ glucose, BGLU - beta-glucosidase, dMAL - D-maltose, dMNE - D-mannose, BXYL - beta-xylosidase, BAlap - beta-alanine arylamidase pNA, PrpA - L-Proline arylamidase, LIP – lipase, PLE – palatinose, TyrA - Tyrosine arylamidase, dTAG - D-tagatose, dTRE - D-trehalose, MNT – malonate, 5KG - 5-keto-d-gluconate, lLATk - L-lactate alkalinisation, AGLU - alpha-glucosidase, SUCT - succinate alkalinisation, NAGA - beta-N-acetyl-galactosaminidase, AGAL - alpha-galactosidase, PHOS – phosphatase, GlyA - glycine arylamidase, lHISa - L-histidine assimilation, CMT – coumarate, BGUR - beta-glucuronidase, O129R - O/129 resistance (comp.vibrio.), GGAA - Glu-Gly-Arg-arylamidase, lMLTa - L-malate assimilation, ELLM – ellman, lLATa - L-lactate assimilation

API 20E: ADH - arginine dihydrolase, TDA - tryptophane deaminase, IND - indole production, VP - acetoin production (Voges Proskauer), GEL – gelatinase, INO – inositol, RHA – rhamnose, MEL – melibiose, AMY - amygdalin, ARA – arabinose

Lab-made: LAC – lactose, SAL – salicin,, XYL – xylose, DUL – dulcitol, , dTAR – d-tartrate, MUC - mucate
